# Supplementary material for: OceanNet: a principled neural operator-based digital twin for regional oceans
Source: Sci Rep. 2024 Sep 11;14:21181. doi: 10.1038/s41598-024-72145-0 (PMC11390968; doi:10.1038/s41598-024-72145-0)
Supplement: Supplementary file 1 — Supplementary Information. [file 41598_2024_72145_MOESM1_ESM.pdf]

Supplementary Information for "A principled neural operator-based digital twin for regional oceans"

Ashesh Chattopadhyay[1*], Michael Gray[2], Tianning Wu[2], Anna B. Lowe[2] and Ruoying He[2*]

[1*]Applied Mathematics, University of California, Santa Cruz, Santa Cruz, 95060, California, United States.
[2]Marine, Earth & Atmospheric Sciences, North Carolina State University, Raleigh, 27695, North Carolina, United States.

*Corresponding author(s). E-mail(s): aschatto@ucsc.edu; rhe@ncsu.edu;
Contributing authors: magray@ncsu.edu; twu27@ncsu.edu; ablowe@ncsu.edu;

1 Performance of an U-NET on the GS

In Fig. S1, we show that a baseline U-NET trained on the same data as OceanNet eventually becomes unstable and show unphysical features in SSH at 120 days.

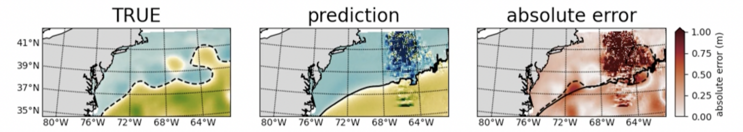

Fig. S1 Prediction performance of a baseline U-NET on the GS region at 120 days.

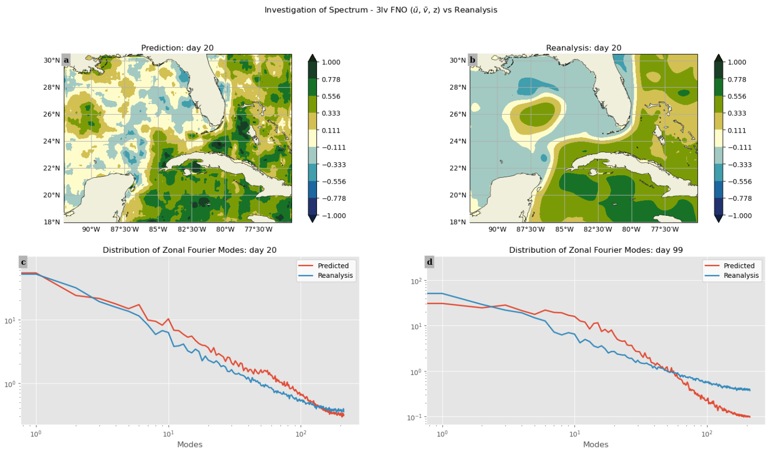

Fig. S2 Demonstration of spectral bias in autoregressive prediction with a simple FNO model without the spectral regularizer and PEC integrator in the GoM. This model was trained on one day lead time with 3 channels: SSH, depth-averaged zonal velocity ($\bar{u}$), and depth-averaged meridional velocity ($\bar{v}$). (a) Prediction after 20 days. (b) Reanalysis data at 20 days. (c) Zonal Fourier spectrum present in (a) and (b). (d) same as (c) but at 99 days.

2 FNO without spectral regularizer or PEC integrator

In Fig. S2, we show that the FNO trained on SSH, depth-averaged zonal velocity $\bar{u}$, and depth-averaged meridional velocity $\bar{v}$, without the spectral regularizer or the PEC integrator. It becomes unstable during autoregressive prediction by day 20 because the high wavenumbers of the Fourier spectrum fail to match the true Fourier spectrum. By day 99, this epistemic error in the small scales affects the accuracy of the large scales as do multi-scale nonlinear interactions in turbulence.

3 FNO with a geostrophic constraint without spectral regularization or the PEC integrator

Several studies in the past have conjectured that certain conservation laws, relevant to the system, may be useful for improving the accuracy of data-driven models [1, 2]. In the FNO model, even without any regularization or PEC integration scheme, the geostrophic constraint–the only relevant conservation law in this system–is satisfied up to $O(10^{-6})$. Hence, conserving geostrophy did not yield fruitful results in this system. The spectral regularizer and the PEC integration scheme was essential to obtain stability in this system.

References

[1] Beucler, T., Rasp, S., Pritchard, M., Gentine, P.: Achieving conservation of energy in neural network emulators for climate modeling. arXiv preprint

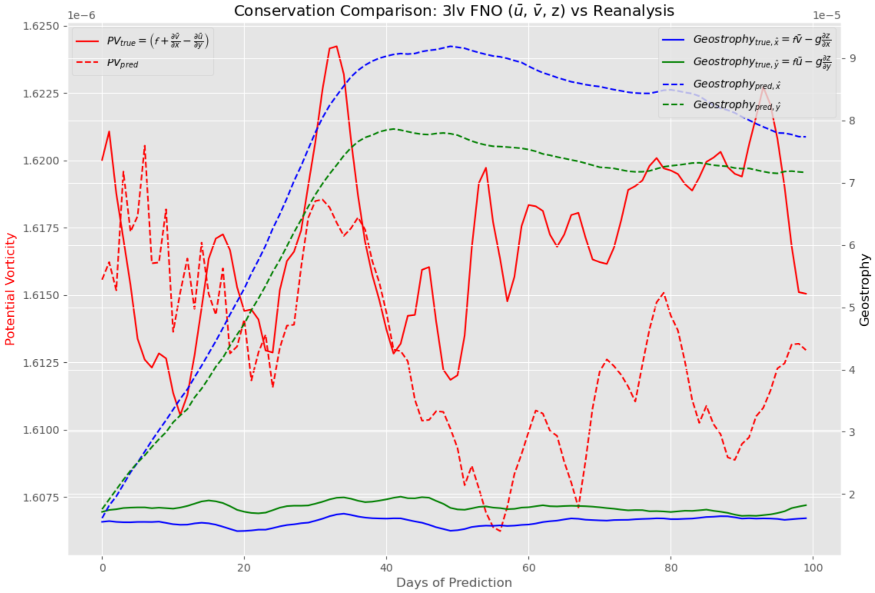

Fig. S3 Comparison of the potential vorticity and the residual geostrophy in the FNO for the GoM region described in section 2. Note the scale of the geostrophy axis is 1e-5 and the scale of the PV axis is 1e-6.

arXiv:1906.06622 (2019)

[2] Beucler, T., Pritchard, M., Rasp, S., Ott, J., Baldi, P., Gentine, P.: Enforcing analytic constraints in neural networks emulating physical systems. Physical Review Letters **126**(9), 098302 (2021)
